# Determinants of vaccine coverage and timeliness in a northern Pakistani village

**DOI:** 10.1371/journal.pone.0263712

**Published:** 2022-02-17

**Authors:** Alexandra F. Jamison, Benjamin J. J. McCormick, Ejaz Hussain, Elizabeth D. Thomas, Syed Iqbal Azam, Chelsea L. Hansen, Zeba A. Rasmussen

**Affiliations:** 1 Division of International Epidemiology and Population Studies, Fogarty International Center, Bethesda, MD, United States of America; 2 Science Fish Limited, Insch, Aberdeenshire, United Kingdom; 3 Administration Department, Karakoram International University, Gilgit, Gilgit-Baltistan, Pakistan; 4 Department of Community Health Sciences, Aga Khan University, Karachi, Sindh, Pakistan; Marie Stopes International, PAKISTAN

## Abstract

The incidence of vaccine preventable disease in Pakistan remains high despite a long-standing Expanded Program on Immunization (EPI). We describe vaccine completeness, timeliness and determinants of coverage from a remote rural cohort (2012–2014). Vaccination histories were taken from EPI records. Vaccination was complete if all doses were received according to the EPI schedule and timely if doses were not ≥3 days early or ≥ 28 days late. Three models are presented: a multivariable logistic regression of household demographic and socioeconomic factors associated with complete vaccination, a multivariable mixed effects logistic regression assessing whether or not the vaccine was administered late (versus on-time), and a mixed effects multivariable Poisson regression model analysing the interval (in days) between vaccine doses. Of 959 enrolled children with full vaccination histories, 88.2 and 65.1% were fully vaccinated following either the pentavalent or DPT/HBV schedules if measles was excluded; coverage dropped to 50.0 and 27.1% when both doses of measles were included. Sixty-four (6.7%) were unvaccinated. Coverage and timeliness declined with subsequent doses. Migrating into the village after 1995 (95%CI 1.88 to 5.17) was associated with late vaccination. Being male, having an older father, and having parents with at least some formal education reduced the likelihood of a late dose. The interval between doses was consistent at 5 weeks (compared with the 4 weeks recommended by EPI). None of the socio-demographic variables were related to the likelihood of receiving full coverage. Vaccine coverage in Oshikhandass was higher than national averages. Measles vaccine coverage and timeliness were low; special consideration should be paid to this vaccine. The local vaccination schedule differed from the EPI, but the consistency suggests good local administration.

## Introduction

The Expanded Program on Immunization (EPI) has dramatically reduced preventable childhood disease through the routine vaccination of children [1, 2]. The success of these programs is, in large part, predicated on achieving near-universal coverage to protect children via vaccination or through herd immunity. Also important is adherence to a schedule of doses to ensure that vaccines are given appropriately. These two characteristics–coverage and timeliness–remain challenges across the world, but especially in resource-constrained populations, where context-specific issues make devising solutions difficult [3].

Childhood mortality in Pakistan, a significant proportion of which is vaccine-preventable [4, 5], has remained high [4] (67.2 deaths of under 5 year olds per thousand live births [6]), in a country that is challenged by both a dispersed, mobile, and sometimes inaccessible population and permeable land borders with other countries that cannot prevent the spill-over of disease. Considerable efforts have been made to understand the relatively low vaccine uptake in Pakistan, in particular driven by coverage of the measles vaccine, especially the second dose, which (introduced in 2009) hovers around 45% [7]; there is also on-going circulation of poliovirus [8], with Pakistan one of two countries with persistent wildtype 1 polio virus in circulation [9]. Reasons for lack of vaccination include: a sparse rural population [10] and the distance to a vaccination center [11, 12]; lack of engagement from doctors and investment in lady health visitors [10, 13]; low levels of parental education [14, 15], in particular the father’s education; animosity toward vaccination [16]; family poverty [14]; and a lack of consistent information about vaccines [11, 12, 17].

Here, we examine the coverage and timeliness of vaccinations in a rural population in Northern Pakistan. Nestled in the Karakoram mountains, Oshikhandass is connected to major cities within Pakistan for work and continued education, and has links with the regional capital Gilgit by the Karakoram highway. The population has undergone substantial change, illustrated by investments in local education that increased maternal literacy from 29.3 to 71.7% (1989 to 2011) [18]. We describe the adherence to the EPI schedule in children under five years, born between 2011 and 2014 following an earlier, long-term cohort study (1989–1996) in the same community that included weekly visits from local health care professionals and promotion of childhood health best practices [18]. We hypothesize that the EPI schedule adherence, following intense health-promotion, was higher than the national average and that families who were enrolled in the original cohort study were more likely to complete their children’s vaccinations than those families who migrated into the community afterward.

## Methods

### Study design

The Water, Sanitation, Health and Hygiene Intervention study (WSHHI) was a community-based observational study measuring childhood mortality and morbidity in Oshikhandass village in Gilgit-Baltistan, Pakistan. It built on an earlier longitudinal cohort study (1989–1996) that examined diarrheal disease and pneumonia in children under five years in the same population. The WSHHI study utilized trained research staff and Lady Health Workers (LHWs), who are healthcare providers trained and paid by the government to provide family health services and education in rural and disadvantaged communities [19]. Between November 2011 and March 2014, all children under 5 years old born in, or migrated into, the village were eligible to be included and there were no exclusion criteria. Every home in Oshikhandass was visited weekly to conduct disease surveillance as well as provide routine healthcare to all children in the village from birth up to the age of five years [18]. Each child in the village was enrolled in the vaccination survey. The final population size was a total sample of all children in the population.

Research staff accessed vaccination records (EPI cards) from mothers and recorded the dates of each child’s vaccinations. If the mother was not in possession of the card, LHW records were consulted and, where necessary, mothers were contacted to fill in gaps. Ethical approval was granted by the NIH NICHD IRB, the AKU Ethical Review Committee and Karakoram International University Ethical Review Committee; the families of all children provided signed consent to participate.

### EPI schedule

Until 2009, the EPI schedule in Pakistan included the Bacille Calmette-Guérin (BCG) vaccine at birth, four doses of the Oral Polio Vaccine (OPV) at birth and 6, 10 and 14 weeks, three doses of the combined diphtheria, tetanus and pertussis (DPT) vaccine at 6, 10 and 14 weeks, three doses of the Hepatitis B (HBV) vaccine at 6, 10 and 14 weeks and a dose of measles-containing vaccine at nine months [20]. The schedule was modified following WHO recommendations in 2009 with the introduction of the pentavalent vaccine to co-administer DPT, HBV, and *Haemophilus influenzae* type b (HiB) vaccines, in three doses at 6, 10 and 14 weeks [20]. However, the pentavalent vaccine was not rolled out uniformly throughout the region, therefore some children still received the DPT/HBV schedule into 2012. In 2009 the protocol added a second dose of measles vaccine to be given at 15 months of age. Most of the children in the study population received free vaccines available through a visiting EPI vaccinator one day each week at the village government dispensary; some children in the study population received their vaccines at private or public clinics in different area towns.

### Household socio-demographic information

At enrolment, families completed surveys about household composition, socioeconomic status, hygiene practices, and questions addressing health literacy. Variables included child sex and birth-order, and the total number of children in the family; educational attainment of both mother and father (no education versus any formal education); average monthly family income and parental occupation; parental age at the time of the child’s birth; and the distance from the house to the vaccine dispensary. A survey of health literacy was administered to mothers that included a question on the causes of illness; this was simplified to whether or not a mother knew that germs were a cause of diarrhea and pneumonia. Since this study followed an earlier study (1989–1996) involving intensive surveillance of the growth and health of children under age five years, as well as a large health education component, families were asked whether they had migrated into the community after that study (*i*.*e*. after 1995), with the assumption that those and hence were less likely to have had exposure to the same health promotion information.

### Statistical analysis

A child was considered fully vaccinated if they received one BCG dose, four OPV doses, two measles doses, and either three pentavalent doses or three DPT and three HBV doses. The analytic sample was limited to those children with a complete vaccine history, or those children who were confirmed to have had no vaccines. If a child’s full history could not be described, they were excluded from the analysis. For schedule adherence, a vaccination was considered timely if it was received no more than 3 days earlier and no more than 28 days later than the EPI schedule. The vaccine interval was modelled as the difference in days between vaccine doses. Two-tailed Kruskal-Wallis (count), t- (continuous) and chi-squared (categorical) tests were used to compare the distributions of variables for participants who did or did not migrate into the village by 1995.

Each of the continuous household and demographic independent variables were centered on their mean value and scaled by their standard deviation; the resultant odds or risk ratios (OR and RR respectively) from models are therefore interpreted as the change in the outcomes for a one standard deviation change from the mean of the respective variable. Missing covariate data were imputed with predictive mean matching [21] 10 times and results were pooled across imputed datasets. Most of the missing values were for family income.

Three models are presented: The first is a multivariable logistic regression examining the relationship between household demographic and socio-economic factors and vaccine coverage. The second model is a Bayesian multivariable mixed effects logistic regression to look at the same household factors and whether a vaccine was administered late. Preliminary analysis showed that it was unusual to have early vaccination (relative to on-schedule or later) and consequently, examination of timeliness was therefore restricted to whether or not a vaccine was late (>28 days after the EPI schedule) or on time and a sensitivity analysis was run to examine a five-week interval rather than the four week interval in the EPI. Two random intercepts were included to account for the repeated observations of each child and for the identity of the vaccines. A term for the dose number was also added to the socio-demographic factors included in the coverage analysis. The third model is a mixed effects multivariable Poisson regression model, constructed to describe the interval (in days) between doses of vaccine. Random intercepts were included for the child and vaccine. All analyses were conducted in R [22] and assumed significance at p≤0.05.

## Results

A total of 1,170 children under the age of 5 were enrolled into the study (median 28.7 months old inter-quartile range 14.0 to 44.5). Of these, 959 had a complete vaccination history with 155 (16.1%) receiving the DPT/HBV sequence and 740 (77.1%) the pentavalent vaccine. Sixty-four (6.7%) children were entirely unvaccinated. Characteristics of the population are shown in **Table 1**. Study children were, on average, the second child born in the family, and families had, on average, 3 children. A quarter of mothers and a tenth of fathers had no formal education. Families who migrated into the village after 1995 tended to have more children, older mothers and both parents were less likely to have formal education, lower incomes and to live further from the dispensary than families that were residing in the village before 1996. Children who were excluded because of incomplete vaccination histories were more likely to have uneducated mothers (63.3% versus 74.3%, p = 0.0013 test of proportions) and were more likely to have migrated into the village after 1996 (33.7% versus 23.8%, p = 0.015 test of proportions).

**Table 1:** Household and demographic characteristics of the population (N = 959 children).

	Migration into village		
	Before 1995	After 1995	*P*
N	722	225	
*median [IQR]*			
Child order	2 [1, 4]	3 [2, 5]	<0.001^2^
Total number of children	3 [2, 4]	4 [3, 6]	<0.001^2^
Maternal age (y)	27 [24, 31]	28 [24, 32]	0.041^2^
Paternal age (y)	32 [28, 36]	33 [28, 37]	0.185^2^
Family income (Rupees)	41169 (85727)	21407 (21303)	0.003^3^
Distance to dispensary (km)	1.07 [0.76, 1.33]	1.14 [0.86, 1.53]	<0.001^2^
*N (%)*			
Sex of child (male)	383 (53.0)	125 (55.6)	0.56^4^
Maternal education (some)^1^	590 (81.8)	114 (50.7)	<0.001^4^
Paternal education (some))^1^	668 (92.6)	187 (83.1)	<0.001^4^
Knowledge of germs as cause of diarrhea/pneumonia (y)	468 (68.4)	121 (58.7)	0.013^4^

^1^Education was classified as some or no formal education

^2^ Kruskal-Wallis test

^3^ *t-*test

^4^ χ2 test

Overall 43.1% (413/959) of children were fully vaccinated, inclusive of children who followed the DPT/HBV and the pentavalent schedules (**Table 2**). Measles vaccine doses were most commonly missed. Excluding the measles vaccine, a total of 653/740 (88.2%) children were vaccinated with all eight appropriate doses in the pentavalent schedules and 101/155 (65.1%) in the DPT/HBV schedule. When the first dose of measles was included, the percentage receiving full coverage dropped to 73.4% and 63.2% respectively for the pentavalent and DPT/HBV schedules, and by the second measles dose, coverage dropped to 50.0 and 27.1% for the two schedules.

**Table 2:** Vaccine coverage and timeliness (N (%)) for the n = 959 children with complete vaccination histories. DPT and HBV (n = 155) were replaced with the pentavalent vaccine (n = 740) part way through the study hence children were eligible for one or other. Timeliness assumes a vaccine should be given within a -3≤ days ≤ 28 window around the EPI schedule.

Vaccine	Dose	Target age	Coverage		Timing		
			Received	Not Received	Early	On Time	Late
					(<-3 days)	(3≤ EPI ≤28)	(>28 days)
BCG	1	Birth	916 (95.5)	43 (4.5)	-	730 (79.7)	186 (20.3)
OPV	1	Birth	853 (88.9)	106 (11.1)	-	687 (80.5)	166 (19.5)
	2	6w	899 (93.7)	60 (6.3)	51 (5.7)	675 (75.1)	173 (19.2)
	3	10w	880 (91.8)	79 (8.2)	28 (3.2)	523 (59.4)	329 (37.4)
	4	14w	847 (88.3)	112 (11.7)	17 (2.0)	344 (40.6)	486 (57.4)
DPT^a^	1	6w	155 (100)	0 (0)	11 (7.1)	104 (67.1)	40 (25.8)
	2	10w	153 (98.7)	2 (1.3)	4 (2.6)	89 (58.2)	60 (39.2)
	3	14w	149 (96.1)	6 (3.9)	2 (1.3)	60 (40.3)	87 (58.4)
HBV^a^	1	6w	124 (80.0)	31 (20.0)	9 (7.3)	87 (70.2)	28 (22.6)
	2	10w	122 (78.7)	33 (21.3)	3 (2.5)	79 (64.8)	40 (32.8)
	3	14w	120 (77.4)	35 (22.6)	1 (0.8)	55 (45.8)	64 (53.3)
Pentavalent^b^	1	6w	740 (100)	0 (0)	43 (5.8)	563 (76.1)	134 (18.1)
	2	10w	722 (97.6)	18 (2.4)	27 (3.7)	426 (59.0)	269 (37.3)
	3	14w	690 (93.2)	50 (6.8)	17 (2.5)	278 (40.3)	395 (57.2)
Measles	1	9mo	738 (77.0)	221 (23.0)	71 (9.6)	353 (47.9)	314 (42.5)
	2	15mo	467 (48.7)	492 (51.3)	249 (53.3)	59 (12.6)	159 (34.0)

^a^ n = 155

^b^ = 740

**Fig 1:**
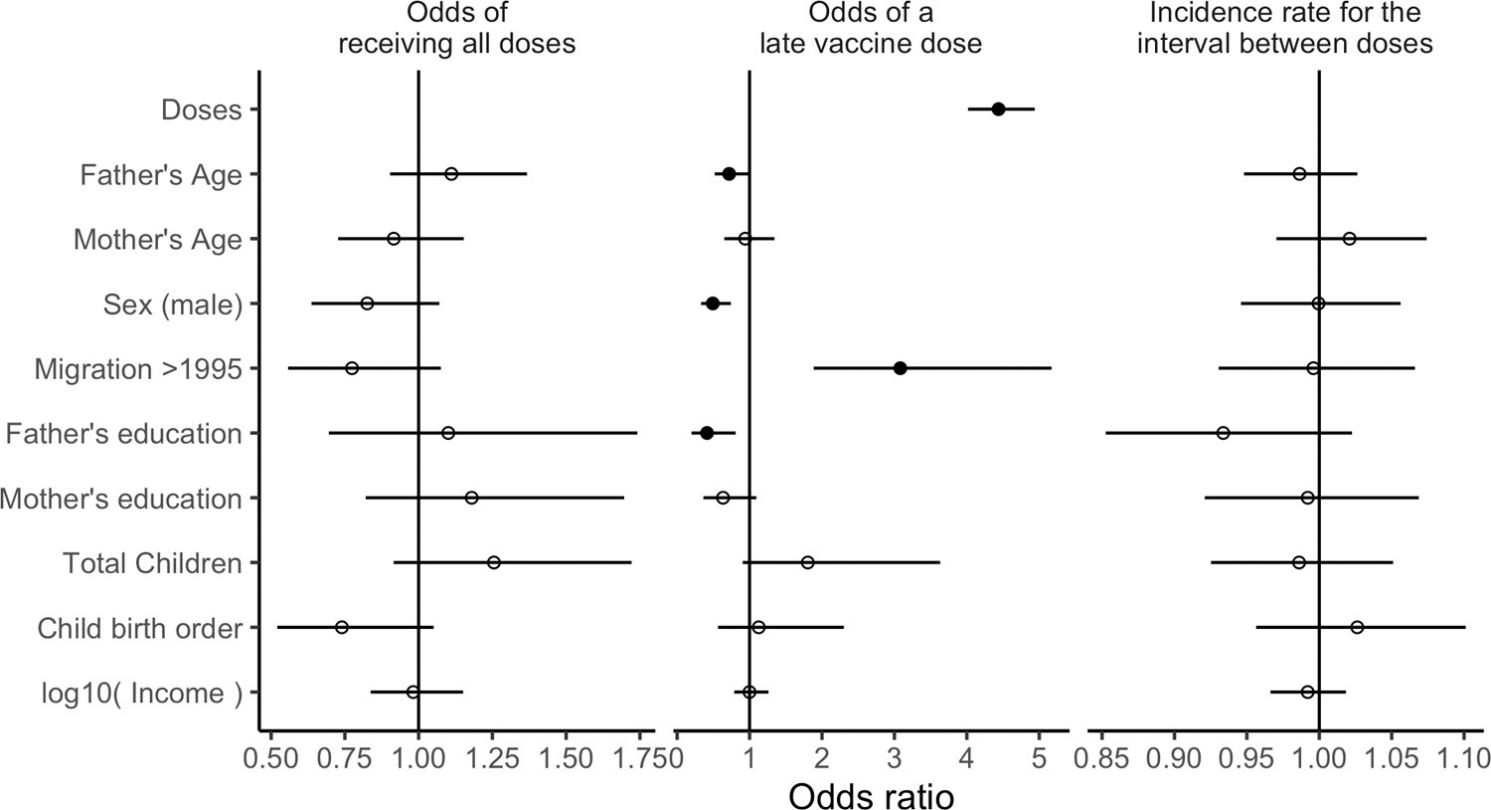
(left) Model predicting the odds ratio of completing the EPI schedule, (center) odds ratio of receiving a vaccine dose late and (right) the risk rate of the interval (in days) between doses. Continuous variables were centred on their mean value and scaled to unit standard deviation; coefficients therefore reflect the difference per one standard deviation from the mean. Solid points indicate that the 95% confidence (or credibility) interval for a coefficient did not include one.

None of the socio-demographic variables were related to the likelihood of being fully vaccinated (at p≤0.05, **Fig 1**). Several factors were, on average, associated with a lower odds of being fully vaccinated: migrating into the community after 1995, higher in the birth order and being male; and other factors that tended to be associated with full vaccination: some formal, parental education belonging to a larger family.

Of all the vaccine doses given, 69.5% were within the window of the EPI schedule (-3≤days≤28, **Table 2**). Few (6.2%, 533/8575) doses were given early and these were mostly the second measles vaccine dose (n = 249). Timeliness declined sharply for later doses of multi-dose vaccines, for example, 80.5% received their first dose of OPV on time, but only 40.6% received a fourth dose on schedule. The likelihood of a late vaccination increased by an odds ratio of 4.44 (95%CI 4.02 to 4.94) for each successive dose. Migrating into the population after 1995 was also a substantial risk factor for late vaccination (OR 3.08, 95%CI 1.88 to 5.17). Being male (OR 0.49, 95%CI 0.33 to 0.74), having an older father (OR per standard deviation over the mean age: 0.72, 95%CI 0.52 to 1.00) and parents with at least some formal education reduced the likelihood of a late dose (OR mother: 0.63, 95%CI 0.36 to 1.09; father: 0.41, 95%CI 0.20 to 0.81). In a sensitivity analysis exploring a five-week interval there were no substantive differences in coefficients (r^2^ 0.95).

**Fig 2:**
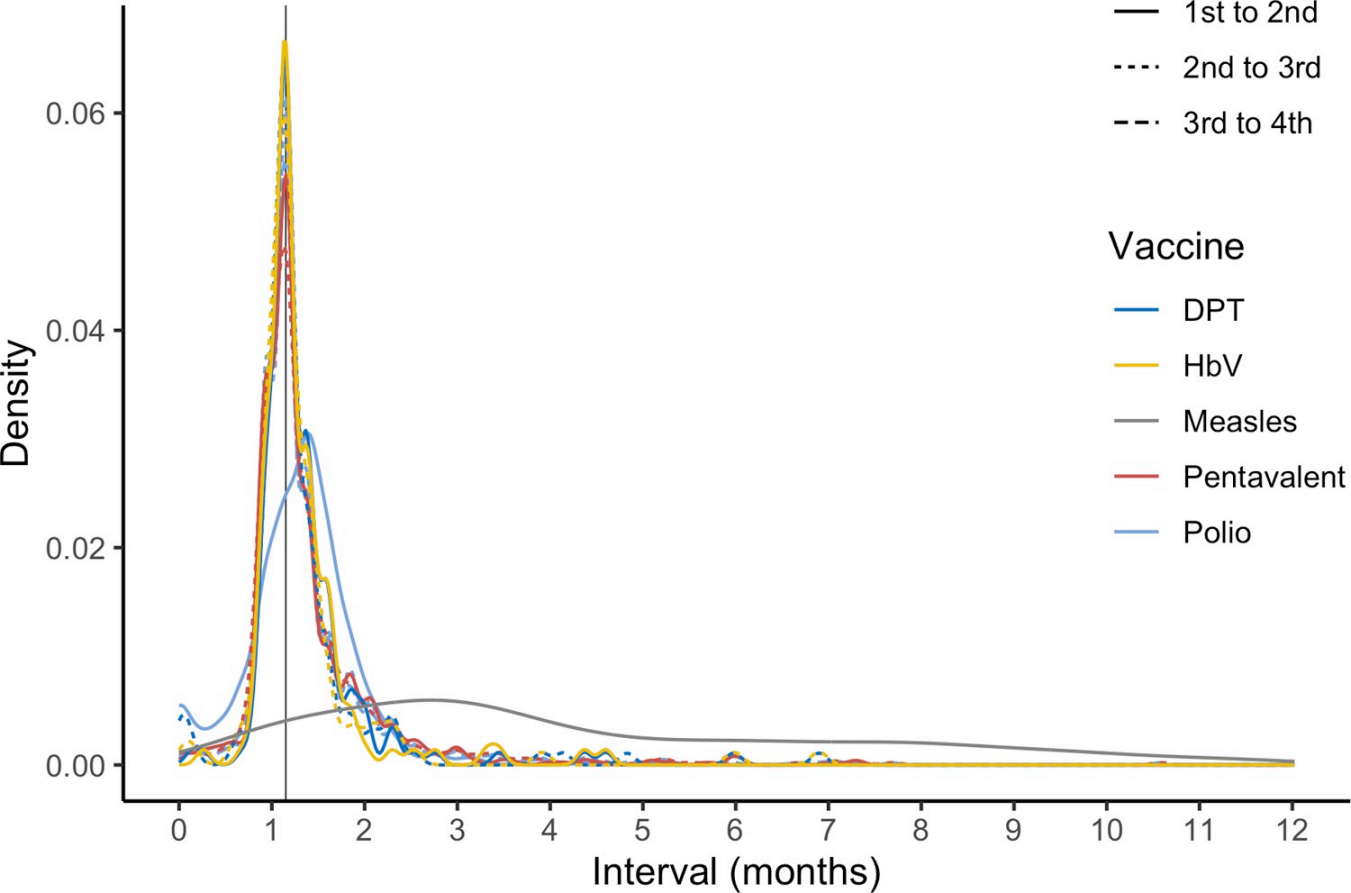
The density of the interval between doses for multi-dose vaccines. The line type indicates which interval (e.g., solid lines indicate the interval between the first and second dose) and the color indicates the different vaccines. The vertical line indicates a five-week interval.

Although subsequent doses tended to be administered later than the EPI schedule, for the first vaccines on the schedule (OPV, DPT, HBV and Pentavalent vaccines) the interval between doses was highly consistent (**Fig 2**). The EPI schedule requires intervals of four weeks for most vaccines (six to 10 to 14 weeks of age). The intervals in Oshikhandass were a median of five weeks, compounding the delay for subsequent doses. The first measles dose was often late (42.5%), and the interval between measles doses was highly variable (median 15.3 weeks, IQR 10 to 30.9 weeks); however, because the interval between measles doses tended to be short, the second dose was often early (**Table 2**). None of the socio-demographic variables had strong signals with the length of interval between doses (**Fig 1**), but the children of fathers with some formal education tended to have shorter intervals between doses (RR 0.93, 95%CI 0.85 to 1.02) than children of uneducated fathers.

## Discussion

Routine vaccination through national EPI programs has dramatically improved the survival of children. In this study, the coverage and timeliness of childhood vaccinations were examined in Oshikhandass, Pakistan. Vaccine coverage in this population tended to be very high (>88% for the BCG, OPV and pentavalent schedule), however that vaccine coverage does not include measles vaccine rates, which were substantially lower than the target levels of 90% national and 80% regional coverage [23]. Vaccines were frequently given late, and increasingly late for each successive dose. However, this was largely due to a local schedule that differed from the EPI and maintained a five-week rather than a four-week interval between most doses. The consequence was an increasing divergence between the official schedule and the observed timing of doses.

There is substantial evidence that LHW-type home-based health service programs improve outcomes for children with pneumonia and help reduce neonatal mortality [10, 24]. In Oshikhandass, LHWs were introduced in 1995, following an intensive child survival and health education program that began in 1989 [18]. Families arriving after 1995 tended to have lower incomes and parental education levels. In this study, migrating into the population after 1995 was associated with a lower chance of receiving all the doses of vaccine. It was also a strong predictor that vaccines were delivered late. Contrary to previous studies that found limited use of vaccine cards [25] and a lack of information for parents to counter misunderstanding about vaccines [12, 26–28], in this population it was uncommon to lack vaccination documentation (18%) and rarer still to have received no vaccines at all (6.7%), suggesting that the LHW were instrumental in promoting vaccination.

When assessing the timeliness of vaccines, it is necessary to draw comparisons with the published EPI schedule, however, here that comparison was misleading. The local schedule, despite being out of synchrony with the national one, was rigorously maintained, suggestive of a robust vaccination system even though an assessment may conclude that vaccines were given increasingly late. It is unlikely that this modest shift in schedule adversely affects the protection afforded from vaccination and modelling of pneumococcal vaccines suggests that the interval between doses has little impact on effectiveness [29]. A likely explanation for the difference between the local and national schedules is the fact that free vaccines were only available on a once-a-week basis by a visiting EPI vaccinator. It is possible that operational and supply issues also contributed to observed delays in vaccination in this rural population; vaccinators were supposed to have access to all required vaccines, but the 20-percentage point discrepancy between DPT and HBV vaccines received (**Table 2**) that should have been received at the same visit suggests that HBV was not always available. For future assessment of the EPI it would be appropriate to assess the interval between vaccine doses to determine whether there is an effective, but locally determined scheduling of vaccines that fails to align with the national EPI but is nevertheless well maintained. It is worth noting that the EPI portfolio continues to grow, for example, since this study the schedule also includes rotavirus (at six and 10 weeks, introduced in 2017) and inactivated polio vaccine (IPV, at 14 weeks introduced in 2015) despite implementation challenges with the existing vaccines. Pneumococcal conjugate vaccine had been introduced in Pakistan in 2012, but the phased introduction meant that it was not in Oshikhandass at the time of the study.

We expected that parental education, like LHW inputs, would be associated with better adherence to the EPI schedule, and this tended to be supported, albeit not strongly. Formal education of either parent, but more strongly for fathers, was associated with getting vaccines on time. In a model of regional vaccine coverage, Imran *et al* [15] found that anything more than primary maternal education (i.e. middle or higher) was associated with having completed the EPI schedule. Other studies have found that parental education is associated with full vaccination [30–32], and education and income tend to be correlated. Thus, we were surprised that in our study household income had no association with any of the three outcomes (completing the EPI schedule, the timing of vaccination or the interval between doses). This finding was also true for Noh *et al* [33], who found inconsistent associations between wealth and vaccination. In contrast, multiple studies have found differences in coverage by wealth quantiles [12, 25, 27, 30], though mostly these studies focused on regions where the income range may be greater; for example, Zaidi *et al* [34] found that only the most extreme comparison between the wealthiest and poorest wealth quintiles showed significantly different DPT and measles coverage.

It was surprising that household socio-demographic features were not more strongly associated with coverage and timing. One reason may be that the local vaccine schedule was rigorously maintained, implied by the very consistent five-week interval between doses. To this end, the timing appeared ‘on paper’ to get later with each successive dose, however, the local schedule would appear consistent and well enforced. This may go some way to explaining why household-level factors were neither significantly nor meaningfully (given the effect sizes) associated with the interval between doses as local immunization drives overcame household differences. Given that this population was the subject of intensive childhood health surveillance much of which involved the same LHWs still operating, there were strong community ties with LHWs. These may account for some of the local successes of routine childhood vaccinations and promotion of similarly well-resourced LHWs could be a template to support timely vaccine acceptance.

The measles vaccination coverage was, however concerning, more so considering that measles vaccine failure can be high in Pakistan [35]. Coverage rates of the other vaccines are higher than national estimates (even if they are lower than WHO targets) [36], *e*.*g*. for 2014, the nationally reported coverage of BCG was 84% (compared to 96% in this population), the first and third doses of DPT are around 80 and 69% (compared to 100 and 96% here) and a third dose of polio vaccine at 70% (compared to 92%). The national coverage for measles-containing vaccines were 71 and 33% for the two doses respectively, suggesting that use of measles-containing vaccines in this population was substantially above the national level for the second dose (49% in Oshikhandass) despite falling short of the target of ≥95%. The second dose of measles vaccine was introduced starting in 2009 and since this study, the national coverage of the second dose of measles-containing vaccine has increased to 58% (as of 2018). Given that the measles doses are given at the oldest ages (12 and 15 months) it may be that children tend to be vaccinated when siblings also receive vaccines, hence the positive association between the total number of children in the family and completing the EPI schedule.

Out of the 1170 children enrolled in the original cohort study, 211 (18%) did not have full vaccination histories available, including children who moved away from the village, and were excluded from the analysis to ensure analytic rigor. Although the study included a survey of sources of health information, there was no specific data on the perceived importance or effectiveness of vaccines, consequently it is unclear in this population how much hesitancy there was or why. While the study recorded routine vaccinations, it is clear from other projects that some children receive many additional doses; for example, Hoest *et al*. [37] found that children living in areas with frequent campaigns received up to 19 doses of OPV. Over the course of this study, the national EPI program organized five polio vaccine campaigns each year, coverage from which was not recorded. Two measles campaigns also occurred during the study period, one in fall 2011 and another in spring 2014. Vaccine cards often only reflect regular doses given at the dispensary; therefore, children whose vaccine cards suggest they have not received a dose or received it late may have in fact received it through these campaigns. The importance of additional doses and timeliness more generally is unclear in the study population. This study was originally designed to investigate diarrhea and pneumonia incidence, and therefore no data were collected on vaccine immune responses (i.e., titres), which hinders interpretation of the timing of vaccination in terms of the protection from infection or interruption to community transmission.

## Conclusions

Vaccine coverage was generally good and higher than national levels in this remote population in rural Pakistan, but coverage for measles was still substantially below international targets to interrupt transmission. Families exposed to regular engagement with community health workers who promoted health education tended to be more likely to adhere to the EPI schedule than families who came into the population later. The local vaccination schedule was different from the national EPI schedule, however, it was rigorously followed, perhaps limited by vaccine availability. Improving vaccine availability and increasing interaction with community health workers have the potential to improve child survival through improved vaccine coverage.
